# IoT-Enabled Real-Time Monitoring of Urban Garbage Levels Using Time-of-Flight Sensing Technology

**DOI:** 10.3390/s25072152

**Published:** 2025-03-28

**Authors:** Luis Miguel Pires, João Figueiredo, Ricardo Martins, José Martins

**Affiliations:** 1 Technologies and Engineering School (EET), Instituto Politécnico da Lusofonia (IPLuso), 1700-098 Lisbon, Portugal; a22311749@mso365.ipluso.pt (J.F.); a22408795@mso365.ipluso.pt (R.M.); 2 Department of Electronical Engineering, Telecommunications and Computers (DEETC), Instituto Superior de Engenharia de Lisboa (ISEL), 1959-007 Lisbon, Portugal; 3 Department of Systems and Informatics (DSI), Setúbal School of Technology, Instituto Politécnico de Setúbal (IPS), Campus do IPS–Estefanilha, 2910-761 Setúbal, Portugal

**Keywords:** time-of-flight (ToF), Internet of Things (IoT), waste management, urban services

## Abstract

This manuscript presents a real-time monitoring system for urban garbage levels using Time-of-Flight (ToF) sensing technology. The experiment employs the VL53L8CX sensor, which accurately measures distances, along with an ESP32-S3 microcontroller that enables IoT connectivity. The ToF-Node IoT system, consisting of the VL53L8CX sensor connected to the ESP32-S3, communicates with an IoT gateway (Raspberry Pi 3) via Wi-Fi, which then connects to an IoT cloud. The ToF-Node communicates with the IoT gateway using Wi-Fi, and after with the IoT cloud, also using Wi-Fi. This setup provides real-time data on waste container capacities, facilitating efficient waste collection management. By integrating sensor data and network communication, the system supports informed decision-making for optimizing collection logistics, contributing to cleaner and more sustainable cities. The ToF-Node was tested in four scenarios, with a PCB measuring 40 × 18 × 4 mm and an enclosure of 65 × 40 × 30 mm. We used an office trash box with a height of 250 mm (25 cm), and the ToF-Node was located on the top. Results demonstrate that the effectiveness of ToF technology in environmental monitoring and the potential of IoT to enhance urban services. For detailed monitoring, additional ToF sensors may be required. Data collected are displayed in the IoT cloud for better monitoring and can be viewed by level and volume. The ToF-Node and the IoT gateway have a combined power consumption of 153.8 mAh

## 1. Introduction

With urban populations rapidly increasing, cities are facing unprecedented challenges in managing waste efficiently. Effective waste management is crucial not only for public health and well-being but also for environmental sustainability and economic development. Traditional waste management systems often struggle with inefficiencies, leading to overflowing bins, increased operational costs, and higher greenhouse gas emissions. These issues underscore the need for innovative solutions to optimize waste collection and reduce environmental impact.

The way waste is managed has a direct impact on the quality of life in cities and on compliance with global environmental commitments, such as the Sustainable Development Goals (SDGs) [1] defined by the United Nations (UN). Among the most relevant SDGs are SDG 11 (Sustainable cities and communities) and SDG 12 (Responsible consumption and production), which encourage a more sustainable and efficient approach to waste management and resource use.

The European Commission has been reinforcing the importance of efficient and sustainable urban waste management, with various deliberations and strategies to promote the circular economy and reduce the environmental impact of cities. The “Circular Economy Package” [2], one of the European Union’s main legislative initiatives, defines a set of targets and measures to improve waste management, including reducing the waste of resources, promoting recycling, and eliminating waste from landfills. This package encourages municipalities to adopt more effective waste management practices that not only improve environmental quality but also contribute to economic development and the creation of green jobs.

The “Strategy for Plastics in the Circular Economy” [3] is another example of how the European Commission is promoting innovative solutions for waste management. This strategy focuses particularly on reducing the use of single-use plastics and improving plastic recycling. In addition, the European Commission has been promoting digitalization and technological innovation in the municipal waste management sector, encouraging municipalities to adopt smart technologies that can optimize waste collection and transport processes. The 2020 “Circular Economy Strategy” [4], for example, highlights the role of technological innovation, including the Internet of Things (IoT), to improve efficiency in waste management, enabling more efficient collection and reducing the environmental impact of urban waste management systems.

The use of IoT technologies to monitor waste bins in real time represents one of the most innovative and effective solutions for urban waste management. Using fill-level sensors, bins can be monitored in real time, allowing collection services to be notified as soon as bins are full. This intelligent management system makes it possible to optimize collection vehicle routes, scheduling collection only when the containers are full. Additionally, it can include an alert mechanism that notifies when bins are nearing capacity, allowing for proactive scheduling of future collection visits to prevent overflow and ensure timely waste management. IoT-based platforms can optimize urban waste collection by minimizing the number of trips made by collecting vehicles, significantly improving the collection of highly toxic waste. Enhancing the security of Smart Waste Management Systems (SWMSs) can also be achieved through holistic models that counter cyber and physical threats. Integrating robotics into intelligent urban waste management, focusing on automation in collection, sorting, recycling, and disposal, can increase operational efficiency and reduce environmental impacts. Systematic reviews of smart containers implemented for sustainability in urban waste management can analyze detection and actuation technologies, providing insights into the effectiveness of various sensor types and mechanisms.

The relationship between smart municipal waste management and SDGs is clear. SDG 11, which promotes sustainable cities and communities, and SDG 12, which promotes responsible consumption and sustainable production, are directly benefited by the implementation of systems such as the one described. These systems promote more efficient waste management and encourage a more rational use of resources, avoiding waste and encouraging recycling. In addition, reducing the environmental impact of urban activities, namely by reducing emissions associated with waste transportation, contributes to the objectives of the Paris Agreement on climate change, which aims to limit global warming to 1.5 °C above pre-industrial levels.

The proposed system, which integrates ToF sensors, a Raspberry Pi, and an ESP32-S3, offers a comprehensive, reliable, and scalable approach to monitoring and data transmission. This solution is particularly suitable for smart city applications due to its cost-effectiveness, scalability, flexibility, real-time monitoring capabilities, reliability, portability, and compact design. The high precision of the ToF sensors ensures accurate measurements, which is vital for effective waste management.

This study aims to demonstrate the possibility of using ToF sensors connected to an IoT network for real-time monitoring of waste bins, through their height (full or empty) and volume. When collection services are notified, via ToF sensors, that bins are full, collection can be scheduled accurately, avoiding unnecessary journeys. The experiment we carried out involved a modified garbage can, mapping it using the ToF sensor (supported by a microcontroller to acquire sensor data), then sending the data via Wi-Fi to a gateway and subsequently to an Internet of Things (IoT) cloud. The results are recorded and displayed.

The article provides a theoretical background on ToF technology, discusses its current state, and references related works in Section 2. Section 3 outlines the materials and methods used in the research. The results obtained are presented in Section 4. Finally, Section 5 concludes the article and offers insights into future work. 

## 2. Background Theory and State of the Art

This section explores the technology behind ToF cameras, starting with an explanation of their structure and operating principles. Following this, we compare ToF cameras with other technologies to highlight their unique advantages. In Section 2.2, we will provide an overview of the latest advancements in the field and delve into relevant research and studies that complement this discussion.

### 2.1. Background Theory

The ToF camera resembles a traditional digital camera, with a lens focusing light on an image sensor, which is a two-dimensional array of photosensitive pixels. However, unlike typical digital cameras, a ToF camera features an active light source to illuminate the scene. The camera captures reflected light from the scene, with each pixel in parallel calculating depth data to create a complete depth map. Light-emitting diodes (LEDs) are commonly used as the light source due to their rapid response time. Most commercial ToF sensors use Near-InfraRed (NIR) wavelengths, around 850 nm, which are invisible to the human eye and allow high reflectance across various materials without interfering with vision [5].

Pixel implementations for image sensors vary according to the operational principle discussed further in the next section. Most current ToF systems are analog, using photodetectors and capacitors to collect and store charge from light pulses before converting it to a digital signal. Fully digital ToF cameras are also under development, using Single Photon Avalanche Diodes (SPADs) that can detect individual photons. Digital ToF systems help reduce noise linked to analog signals and the conversion process [5]. SPADs are specialized photo detectors offering high sensitivity to low light, capable of detecting single photons. Operating in avalanche mode—biased above their breakdown voltage—SPADs amplify each detected photon into a cascade of charge carriers, creating a detectable current pulse. This amplification enables SPADs to sense extremely faint light levels, down to individual photons [6].

The timing precision of SPADs is critical for applications like ToF sensors and Light Detection and Ranging (LiDAR), where accurate distance calculations depend on detecting light pulses. By measuring the time delay between a photon’s emission and its return after reflecting from an object, SPADs calculate distances with high temporal resolution. This capability is especially valuable in robotics, 3D scanning, and automotive sensing, where detailed depth information is crucial. However, SPADs have some limitations, such as after pulsing, where a SPAD remains sensitive after detecting a photon, potentially causing false counts, and dead time, which limits the detection rate, especially in highly lit or rapid-detection environments [5,6].

The fundamental operating principle of ToF cameras involves illuminating a scene with a modulated light source and measuring the light that reflects to the sensor. Since the speed of light is constant, the distance to the object from which the light was reflected can be determined by calculating the time difference between the emitted and returning light signals. ToF cameras use two primary illumination techniques: pulsed light and Continuous Wave (CW) modulation.

In pulsed light ToF, short bursts of light are emitted, and the time taken for the light to return is measured to calculate distance. In CW modulation, the light source emits a continuous, sinusoidally modulated wave, and the phase shift between the emitted and received light is used to compute the depth information.

In the pulsed method, depth measurement is straightforward. The illumination unit quickly switches on and off, creating short pulses of light. A timer is activated when the light pulse is emitted, and stops when the reflected light is detected by the sensor. The distance, *d*, to the object, is then calculated based on the elapsed time, as described in [7].

(1) $$d=∆t\cdot \frac{c}{2}$$

where ∆*t* is the round-trip time of the light pulse and *c* is the speed of light. However, the ambient illumination usually contains the same wavelengths as the light source of the ToF camera. The light captured by the camera consists of both emitted light and ambient light. This mixture can cause inaccuracies in calculating distances. To address this, a measurement is taken when the illumination unit is switched off, allowing the ambient background to be subtracted from the overall signal. This adjustment is managed by using the outgoing light signal as a control for the sensor detector.

Additionally, each short light pulse contains a relatively low amount of energy [6], and due to imperfections in the system components [8], the signal received is prone to noise. To improve the signal-to-noise ratio (SNR), multiple cycles of these pulses—often millions—are recorded over a specific period. The final depth information is derived from the average of these cycles. This recording period is known as the Integration Time (IT) [9].

**Figure 1:**
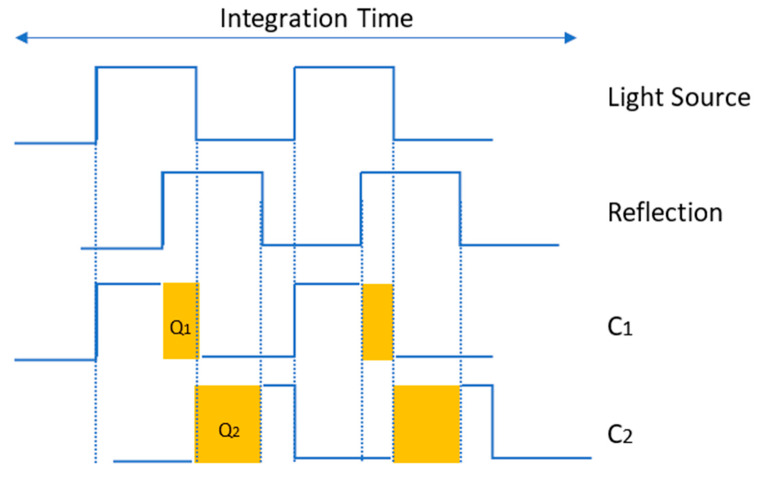
Pulsed ToF method: The received signal is sampled in two out-of-phase windows in parallel (adapted from [9]).

Figure 1 illustrates the concept of this pulsed modulation method. By using an integration time interval of ∆*t* and two sampling windows (C_1_ and C_2_) that are out of phase, the averaged distance can be calculated [9].

(2) $$d=∆t\cdot \frac{c}{2}\cdot \frac{Q_{2}}{{Q_{1}+Q}_{2}}$$

where *Q*_1_ and *Q*_2_ are the accumulated electrical charges received over the integration time.

The depth resolution achievable with the pulsed method is constrained by the speed of the camera’s electronics. Based on Equation (1), achieving a depth resolution of 1 mm would require a light pulse lasting approximately 6.6 picoseconds. However, the rise and fall times, as well as the repetition rates of current LEDs and laser diodes, impose practical limitations on generating such short pulses [8]. Moreover, reaching these speeds in the receiver circuit is challenging with today’s silicon-based technology, especially at room temperature [9].

In the Continuous Wave (CW) method, instead of directly measuring the round-trip time of a light pulse, the CW modulation method determines the phase difference between the sent and received signals. In this approach, the light source is modulated by adjusting its input current, creating a waveform signal [8]. While various modulation shapes can be used, square or sinusoidal waves are the most common [10]. The CW modulation technique reduces the requirements for the light source, enabling a finer depth resolution than is possible with pulsed light.

**Figure 2:**
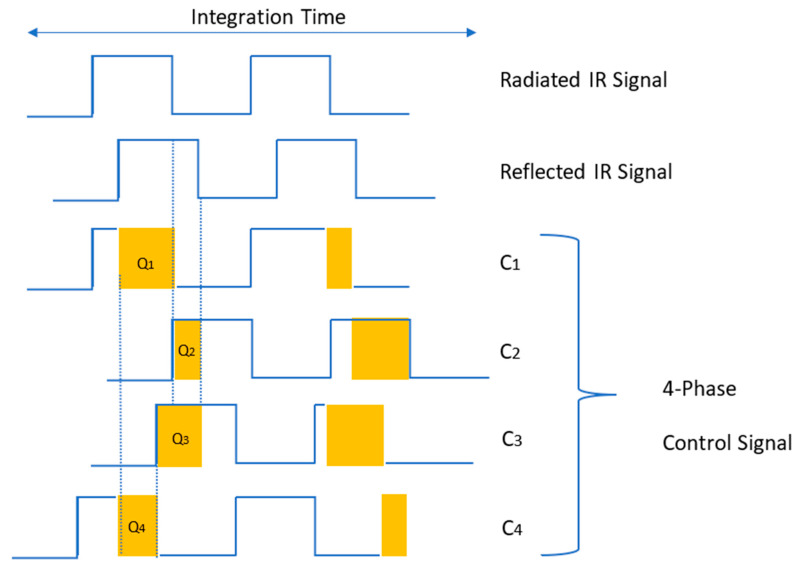
Demodulation principle of CW method: C1 to C4 are control signals with 90° phase delay from each other (adapted from [9,11]).

There are multiple ways to demodulate the received signal and extract its amplitude and phase information. A traditional approach involves calculating the cross-correlation function between the original modulation signal and the returned signal [10]. This cross-correlation can be obtained by measuring the returned signal at specific phases, which can be implemented using mixers and low-pass filters in the detector. A more efficient alternative is synchronous sampling, where the modulated returned light is sampled simultaneously with a reference signal at four different phases (0°, 90°, 180°, and 270°), as shown in Figure 2 [10]. This synchronous sampling approach simplifies the circuit design and reduces pixel size, allowing for more pixels on a sensor and thus higher resolution.

Like the pulsed method, multiple samples are recorded and averaged to improve the signal-to-noise ratio (SNR). By using four equally spaced sampling windows (*Q*_1_ to *Q*_4_), timed by the reference signal (see Figure 2), the received signal is sampled at different phases over the integration time. Assuming a sinusoidal modulation signal with no harmonic frequencies, Discrete Fourier Transform (DFT) equations can be applied to calculate the phase *ϕ*, amplitude *A*, and offset *B* as follows [9,10]:

(3) $$\emptyset =\tan^{-1}\left(\frac{Q_{3}-Q_{4}}{Q_{1}-Q_{2}}\right)$$

(4) $$A=\frac{\sqrt{{\left(Q_{1}-Q_{2}\right)}^{2}+{\left(Q_{3}-Q_{4}\right)}^{2}}}{2}$$

(5) $$B=\frac{Q_{1}+Q_{2}+Q_{3}+Q_{4}}{4}$$

From the phase, the distance can be finally calculated as [9]:

(6) $$d=\frac{c\cdot \emptyset }{4\cdot \pi \cdot f}$$

The intensity, i.e., amplitude *A*, of the light decreases proportionally to the traveled distance in a known way. Hence, the received amplitude value from (4) can be used as a confidence measure for the distance measurement. Additionally, the reflected signal is often superimposed with background illumination, which causes errors in the measurement. Thus, the offset (5) is used to distinguish the modulated light component from the background light [10].

When calculating distances from the phase difference as in (6), one important thing must be considered. Since the modulation signal is periodical, its phase wraps around every phase.

### 2.2. Relevant Research and Studies

According to [12], it proposes IoT-based platforms addressing urban waste collection optimization. Here, the idealization resorts to maximizing the minimizing number of trips made by collecting vehicles for collecting toxins. Algorithms are based on knapsack methodologies. The model was embedded in MATLAB 24.1, and the output derived was a 47 percent improvement in the collection of highly toxic waste as compared to other conventional procedures. Through waste transportation optimization techniques, the waste collection points are established in the cities and are being outfitted with IoT sensors. These IoT sensors can monitor the waste volume and its state of toxicity. The containers are further classified into three groups according to toxicity: high, medium, and normal. The IoT sensors—ultrasonic and gas by default—will collect data on how full a container is and a toxicity level of 2 in the waste. The authors employ the 0/1 knapsack algorithm to optimize the collection trucks loading, so that maximized capacity is ensured, with priority for highly toxic waste being loaded. This model was evaluated in comparison with three traditional methods: First Bin First (FBF)—Collection based on location; Largest Bin First (LBF)—Fullest containers prioritized; and Longest Delay (LD)—Containers that have waited the longest prioritized. In a simulation environment, the following results were presented by the authors: 47% improvement over traditional methods in highly toxic waste collection; fewer trips made by a collection truck; reduced operating costs and fuel consumption; speedier disposal of toxic materials in the optimization of priority waste collection; and a cost—benefit analysis claiming that the system could recover the original investment in sensors in less than one year.

One paper [13] examines the cyber and physical threats affecting Smart Waste Management Systems (SWMSs). The authors mention that the security of SWMSs largely entails securing their communication only while ignoring the vulnerabilities within their physical components and operational infrastructure. The authors introduced a holistic security model aimed at countering cyber and physical attacks. The methodology presented in the article includes observing existent SWMS implementations, their components, architectures, and protocols. With respect to the STRIDE model of identifying vulnerabilities in various layers of SWMSs (spoofing, tampering, repudiation, information disclosure, denial of service, elevation of privilege), an analysis was done on the assessment of possible attacks and their implications covering sensor and communication failures, storage infrastructure, and surveillance. Analysis showed that the SWMSs have adopted promising technologies, starting from sensors measuring the filling level (ultrasonic sensors) to gas and temperature detection devices. The communication networks used for comparison are Long Range Wide Area Network (LoRaWAN), ZigBee, Wi-Fi, and GSM, each with its vulnerabilities. The anomalies in the containers are detected through surveillance cameras monitoring them. All the data are being managed in the cloud, entailing remote access and predictive analysis. Some of the major findings include vulnerabilities in IoT sensors that allow various attacks like spoofing data and falsifying container status; possible communication failures, including Denial of Service (DoS) attacks that can cause damage to data collection activity and operation of SWMSs; threats to user privacy where possible tracking and profiling can be carried out based on the data being collected from smart containers; weaknesses in actuator protection making it possible for different attacks such as blocking the opening of containers or tampering with waste compactors; and security recommendations including strong encryption, strong authentication, and physical protection against device tampering.

Another study [14] presents an account of the influence pertaining to robotics on intelligent urban waste management, with a focus on automation in collection, sorting, recycling, and disposal. The paper reviews existing and emerging technological solutions while examining the integration of robotics, IoT sensors, and AI to enhance operational effectiveness and reduce environmental impacts. Applications such as robotic compactors, autonomous recycling vehicles, and drone monitoring systems are considered. The authors of the paper use robotic technologies for automatic waste collection, including autonomous vehicles integrated with computer vision and AI and ultrasound sensors for monitoring the fill levels of containers and waste categorization, and robotic compactors to maximize waste volume. Drones are used in waste monitoring to detect illegal dumping and optimize collection routes. The study concludes that integrating robotics into urban waste management enhances the efficiency of operations, decreases operational costs, and reduces human exposure to hazardous environments. AI helps optimize collection routes and improve the separation of recyclable material, thus enhancing recycling rates and minimizing waste quantities in landfills. Nonetheless, further studies are required to assess the technology’s economic viability and its potential long-term environmental impact.

The systematic review present in article [15] deals with smart containers implemented for sustainability in urban waste management. Detection and actuation technologies used in those systems were analyzed for their capability of waste segregation. The work has been developed in line with the Systematic Literature Review (SLR) protocol based on the PRISMA methodology with regards to transparency and replicability. The process includes three main stages, such as definition of the need for the study, formulation of the research questions, and selection of the IEEE Xplore, Scopus, and ACM databases; application of inclusion/exclusion criteria and data extraction using reference software; narrative and thematic analysis of the extracted data.

The study identifies the technologies that are used in smart containers, e.g., the most used sensors are “Filling level” (84%)—Predominantly ultrasonic voltage detectors; “Gas detection” (18%)—Gases, including CO_2_ and methane, have been monitored; “Environmental Sensors” (19%)—Measurement of temperature, humidity, and pressure; “Weight sensors” (15%)—Use of weight cells for assessing the weight of waste; “Computer vision” (23%)—Neural network algorithms for classifying waste; “RFID” (3%)—Identifying the waste or users.

Regarding actuators, the report states that there are lid control (28%); automatic motorized mechanisms to work without contact for routing wastes (34%); and automatic rotary and gravity mechanisms for separation and waste compaction (6%) for increased capacity storage. As to the remarks, however, the study found out that there are very few widely adopted detection mechanisms for smart containers. On the contrary, waste classification is still focused on computer vision, having little exploration into composition identification methods. Most automatic waste separation solutions are still very basic and lack standardization. But the report recommends conducting market studies and cost–benefit analysis to set up new high-end sensors for sorting and monitoring at the container level. This last bolsters the requirement and importance of our work with the ToF sensor being specified.

Article [16] provides a comparative analysis of ToF and LiDAR sensors specifically for indoor mapping applications. The primary objective of the study is to assess the accuracy, efficiency, and suitability of ToF sensors in relation to conventional LiDAR systems for indoor use. The research methodology involved conducting a series of experimental tests within indoor settings, utilizing both sensor types to gather spatial data. Key parameters evaluated included the accuracy of distance measurements, spatial resolution, response time, and the capability to detect objects under varying environmental conditions. Findings indicated that LiDAR sensors deliver superior accuracy in distance measurement, with an average error margin of approximately 2 cm, whereas ToF sensors exhibited an average error of about 5 cm. Nonetheless, ToF sensors were noted for their cost-effectiveness and lower energy consumption, rendering them a viable option for applications where utmost precision is not essential. Furthermore, ToF sensors proved to be more effective in identifying objects in highly reflective environments, a scenario where LiDAR systems may encounter challenges due to signal saturation. In conclusion, the decision to utilize either ToF or LiDAR sensors for indoor mapping should consider the necessary accuracy, associated costs, and the specific characteristics of the application environment.

Table 1 summarizes the relevant research discussed previously.

In this study, we aim to demonstrate that while some of these solutions may offer accuracy comparable to our ToF-Node (e.g., LiDAR) or similar features and optimization capabilities (e.g., RFID or IoT-based systems), our system provides a balanced combination of accuracy, cost-effectiveness, and easy implementation. This makes ToF sensors a strong contender for waste bin monitoring applications.

Based on the related works that have been analyzed, we conclude that, while LiDAR offers the highest accuracy and range, it is more expensive and complex. So, it is not a good solution for this type of application. RFID and IoT-based systems provide advanced features and optimization capabilities (like our system) but tend to be more costly and complex to implement. ToF sensors, being inherently low-power (without the need for complex algorithms to achieve this), offer a practical and efficient solution for monitoring trash bins. Their high accuracy ensures precise fill level measurements, and their low power consumption makes them more autonomous, which is essential for this application, especially in scenarios where cost and simplicity are key considerations.

Our research contributes to the field in several significant ways that are not covered by the works referenced previously, namely:

- Precision and Accuracy
- Three-Dimensional Visualization
- Future Potential with Blockchain
- Cost-Effectiveness and Scalability
- Real-Time Monitoring and Reliability

In Section 4.4, we will further elaborate on this topic.

## 3. Materials and Methods

This section is divided by subsections and provides a concise and precise description of the experimental system, and descriptions of the different scenarios, hardware integration, and algorithms developed for the gateway and node.

### 3.1. System Implementation and Hardware Setup

**Figure 3:**
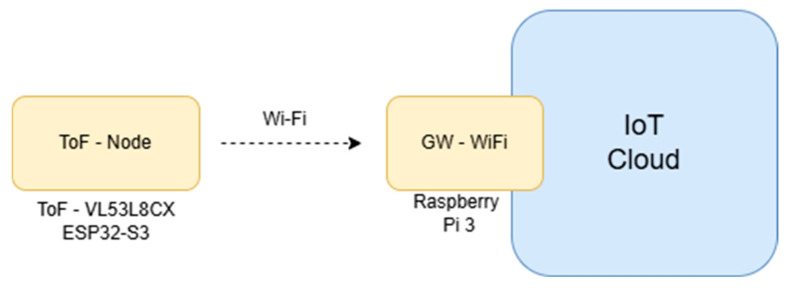
System architecture.

Figure 3 presents the system architecture for the experiment. The system is composed of the ToF sensor—VL53L8CX (STMicroelectronics, US) [17] and the ESP32-S3 (Espressif Systems, China) [18]—these two elements are the named the ToF-node; we developed a Printed Circuit Board (PCB) with these two elements. Outside of the ToFNode, we have a Wi-Fi gateway based on a Raspberry Pi 3 model B (Raspberry Pi, UK) [19] and an IoT Cloud [20].

The VL53L8CX sensor, developed by STMicroelectronics [17], is a distance measurement module that utilizes ToF technology, employing Continuous Wave (CW) modulation for high-precision distance measurements. This sensor is favored for its capability to measure distances across multiple zones concurrently and its durability in various environmental conditions. It incorporates a Vertical Cavity Surface Emitting Laser (VCSEL) that emits infrared light at a wavelength of 940 nm. This emitted light reflects off surrounding objects and returns to the sensor, where it is detected by an array of SPADs. The sensor calculates the time of flight of the light using CW modulation, which facilitates the measurement of the phase of the reflected light to ascertain distance. CW modulation allows the sensor to differentiate between various light paths (both direct and reflected), thereby minimizing errors induced by ambient light or multiple reflections. This technique enhances both accuracy and spatial resolution, enabling dependable measurements even in challenging environments.

**Figure 4:**
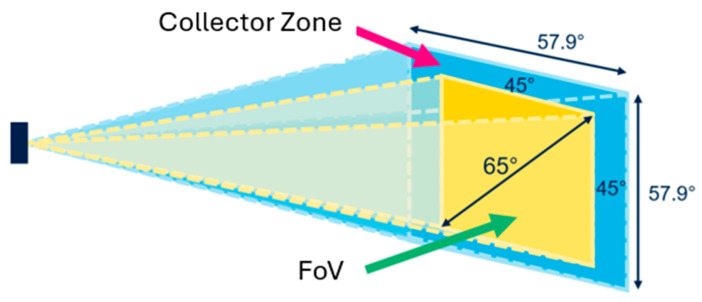
Projection angle and diagonal FoV (adapted from [9]).

The VL53L8CX sensor features sophisticated processing capabilities to mitigate the effects of ambient light and interference. It supports multizone measurements, allowing for distance measurements in up to 64 simultaneous zones arranged in an 8 × 8 matrix, with adjustable spatial resolution ranging from 4 × 4 to 8 × 8 zones based on specific application needs. The sensor’s operational range extends up to 4 m, contingent upon the object’s reflectivity, with a minimum measurable distance of 1 cm and an accuracy of ±1 cm. The Field of View (FoV) can be configured to a maximum of 63° × 63° (Figure 4), and the update frequency can reach up to 60 Hz (for 8 × 8, frequency: 15 Hz and 4 × 4, frequency: 30 Hz). Communication with the microcontroller is facilitated via an I2C interface operating at a voltage of 3.3 V. The sensor’s dimensions are 4.4 mm × 2.4 mm × 1.9 mm.

**Figure 5:**
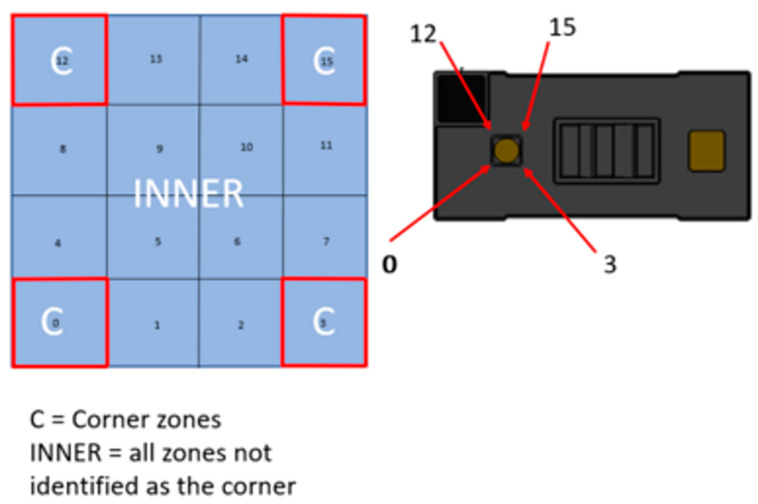
ToF sensor for 4 × 4 resolution (adapted from [9]).

**Figure 6:**
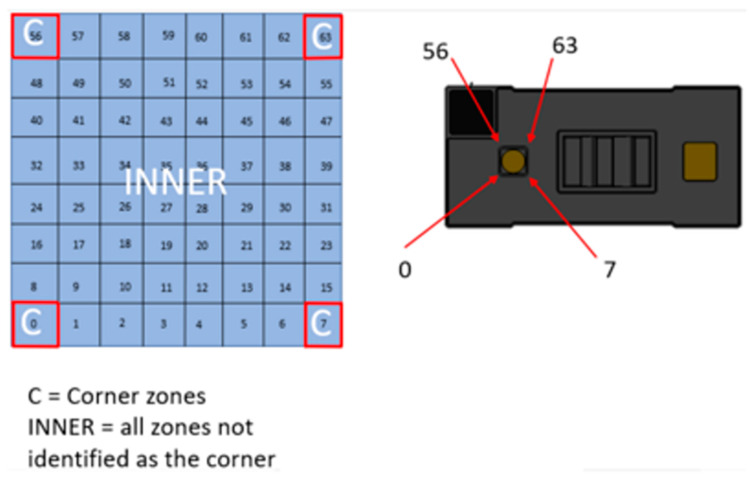
ToF sensor for 8 × 8 resolution (adapted from [9]).

In Figure 4, we have a 45° projection angle with a 65° diagonal FoV. There are potential problems (Figure 5 and Figure 6): the sensor has different resolutions, as we know, and this is going to affect our values; for instance, the values of the corners are the most inconstant, the inner ones being the ones we want to work with, so, using the 4 × 4 resolution, we have around 12 points that we can work with, whilst the 8 × 8 resolution gives us 60 points.

When we combine these 2 topics (range and reflection) into a table to compare values (4 × 4 vs. 8 × 8), we get a better understanding about what we are working with and possible problems that we might face. Ref. [9] uses a table with: FoV, zone and ambient light for the 8 × 8 resolution. 

The ESP32-S3 [18] is a microcontroller with a dual-core processor running up to 240 MHz, featuring an ultra-low-power RISC-V co-processor. It supports Wi-Fi (2.4 GHz) and Bluetooth 5 (including BLE and Mesh). Its compact size (23.5 mm × 18 mm) and integrated ceramic antenna make it ideal for space-constrained applications. In this setup, I2C pins are connected to a ToF sensor.

The Raspberry Pi 3 Model B [19] is a single-board computer running Ubuntu Server 22.04.4 LTS on a 32 GB micro SDHC card (SanDisk Corporation, Milpitas, CA, USA). In this experiment, it acts as a gateway between the ToF-Node and the IoT cloud (Sensefinity cloud [20]).

### 3.2. Test Scenarios

In this experiment, a hollow block was used to establish the basic reference height. This was crucial for the study, as it provided an understanding of the initial depth at which we were working, serving as a useful starting point for analysis. This scenario was tested using the same blocks as a base (36 mm), with two additional blocks of 36 mm height placed on top of the original, central object. This setup aimed to observe the effect of adding structural elements on the measurements made by the sensor.

A more complex scenario involved combining multiple structure objects that were superimposed. This scenario focused on measuring heights in intricate ways, finding exact locations of the holes, and pinpointing high or low points. Factors such as the color of the object, angle of slant, room lighting, and potential sensor errors were considered to enhance the accuracy and reliability of the measurements.

In the final scenario, the objective was to analyze the measurements taken based on a remote setup. Here, the sensor collected data and transferred them to a cloud platform. The data processing from the sensor included dimensions of objects, depth measurements, hole locations, and surface variations. These data were processed and compared in a digitally designed environment for the following:

1. Dynamic analysis on these measurements once processed in the cloud to rapidly adapt, identify patterns, and draw conclusions.
2. Integration of the results with predictive models or algorithms within substructures, correlating factors with brightness.

### 3.3. Hardware Integration

**Figure 7:**
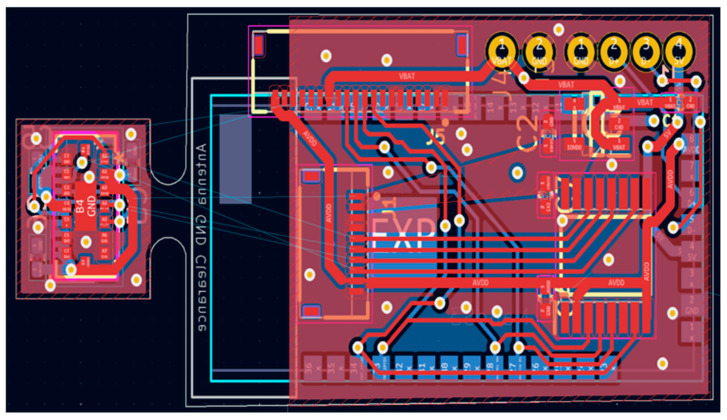
PCB layout of the ToF-Node: ToF sensor and the ESP32-S3.

**Figure 8:**
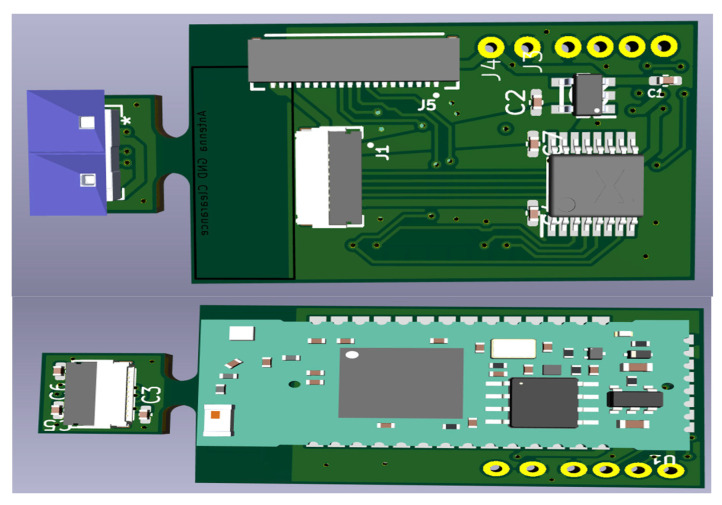
ToF-Node 3D view: PCB with ToF sensor and the ESP32-S3.

Based on Figure 3, and for more facility during the experimental test, we designed a schematic and, after this, a PCB layout. Figure 7 shows a PCB layout that includes a ToF sensor and the ESP32-S3. The PCB has 40 × 18 × 4 mm dimensions. Figure 8 shows a 3D view (top and bottom).

**Figure 9:**
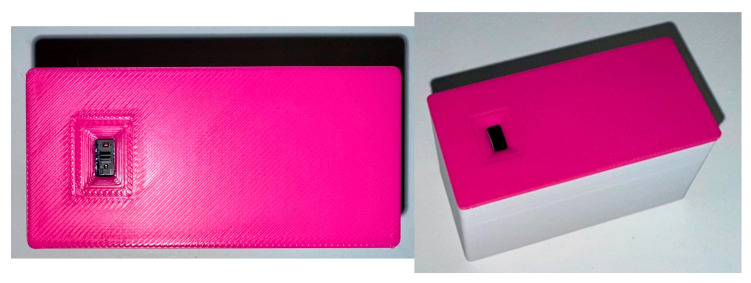
Final prototype (ToF-Node) for use in the experiment.

**Figure 10:**
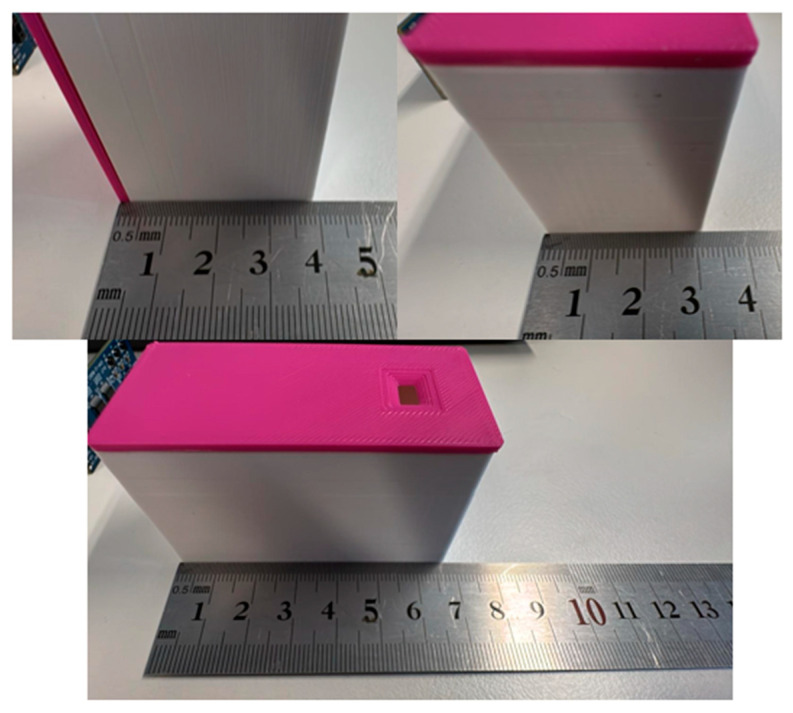
Final prototype (ToF-Node), with dimensions.

The final prototype for the experiment, with the components assembled inside a box and prepared for the use in the experiment; Figure 9 and Figure 10 (with dimensions).

### 3.4. Algorithms Developed

**Figure 11:**
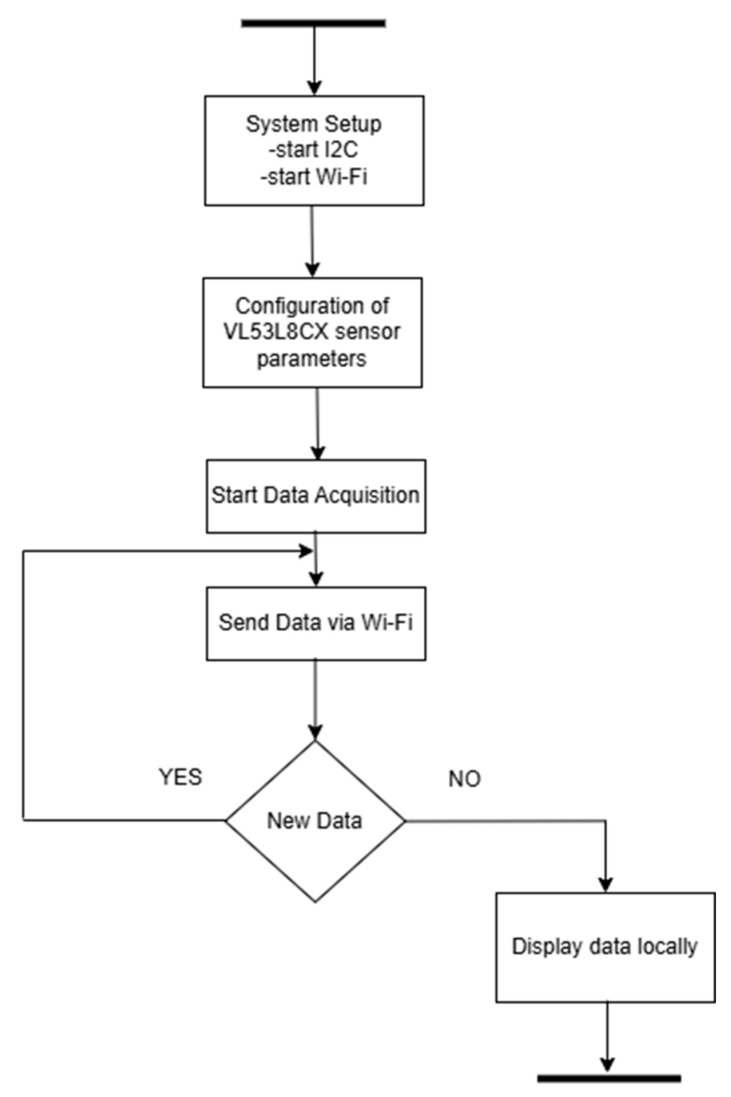
Flowchart of the ToF-Node.

In the ToF-Node, a program was developed for the ESP32-S3 board in C/C++, whose main function is to take the sensors reading and create a payload with these values to be sent to the Wi-Fi and show the values locally. The flowchart is present in Figure 11, and Algorithm 1 is shown after flowchart.
**Algorithm 1**: Integration of the ToF-Node with Wi-Fi     1. System Setup       1.1. Initialize the I^2^C communication bus.       1.2. Initialize the wireless communication module (ESP32-S3).       1.3. Enable the power pin for the sensor, if available.       1.4. Configure the VL53L8CX sensor with the following parameters:           -Resolution: 8 × 8 zones, Set to VL53L8CX_RESOLUTION_8X8           -Metrics Enabled:               Signal Intensity (Signal): Disabled by default               Ambient Light (Ambient): Disabled by default       1.5. Start data acquisition from the sensor.     2. Main Operation Loop       While the system is running:       2.1. Check if new data is available from the sensor.           -If new data is ready:              (a) Retrieve distance measurements for all 64 zones (8 × 8).              (b) Retrieve additional metrics: signal intensity and ambient light.              (c) Format the data for transmission.              (d) Transmit the data to the remote device via Wi-Fi.     3. Data Display (only for monitoring ToF-Node locally)       3.1. Organize and format measurement data.       3.2. Display the data by zones, including distance, signal intensity, and ambient light values.

The Raspberry Pi was set up to connect to a Wi-Fi network, functioning as a gateway with cloud access. It was configured to operate as a Hypertext Transfer Protocol (HTTP) server, utilizing a Representational State Transfer (RESTful) API that facilitated communication with external devices. Through this API, the Raspberry Pi could receive data transmitted by the ESP32. Additionally, the Raspberry Pi had internet access, which was essential for forwarding the received data to the cloud, thereby enabling communication between the local device (ESP32) and the remote cloud infrastructure.

The ESP32-S3 was also configured to connect to the same Wi-Fi network as the Raspberry Pi. It was programmed to gather local data, such as that from a ToF sensor, and to periodically transmit this data to the Raspberry Pi over Wi-Fi. The transmission frequency was set so that the ESP32 would send information either at regular intervals or triggered by specific system events, such as sensor readings.

In this communication framework, the ESP32-S3 functioned as a Wi-Fi client, establishing a connection to the network already linked to the Raspberry Pi. The Raspberry Pi served as a server, awaiting connections from the ESP32-S3. To transmit data, the ESP32-S3 employed HTTP requests, leveraging a RESTful API for interaction with the Raspberry Pi. The data were sent via a GET request, with the information included as parameters in the request Uniform Resource Locator (URL). This method of GET request allowed for the efficient transmission of small data volumes, utilizing the URL to convey information as parameters.

Upon receipt of the data, the Raspberry Pi undertook the processing of the information. This process involved converting the received data into an appropriate format, such as JSON, to ensure ease of manipulation and compatibility with cloud systems. Once the data were processed, they were prepared for transmission to the cloud.

The data were sent to the cloud using the HTTPs protocol, which ensured the security of the data transmission. The Raspberry Pi initiated HTTPs requests to a RESTful API hosted in the Sensefinity cloud [20].

## 4. Results

This section is divided by subsections: firstly, applying the ToF-Node in different scenarios (with augmented complexity) and a cloud scenario. In Section 4.3, we write about the energy consumption of the ToF-Node.

### 4.1. Experiment Scenarios

The following paragraphs explain the two first scenarios defined for the experiment, where the ToF-Node was at a height of 25 cm. All the results presented in this section show that the matrix functions as a “depth map” of the scene in front of the sensor, with the numbers in the cells representing distances in millimeters. The 8 × 8 format indicates that the sensor is taking multiple measurements in a two-dimensional matrix, where the differences in values reflect the variation in depth, with smaller values indicating proximity and larger values representing longer distances.

**Figure 12:**
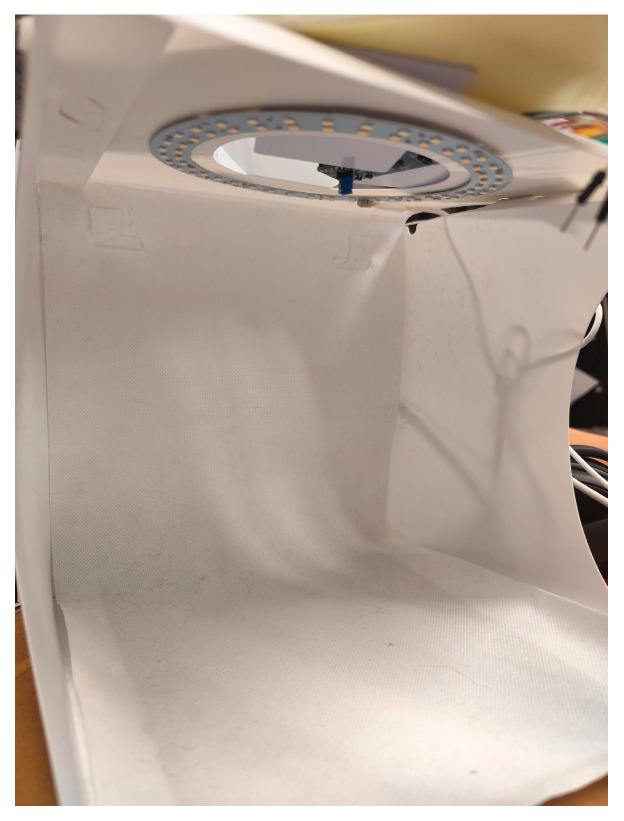
Office trash box—main “scene” for tests (inside and profile view).

**Figure 13:**
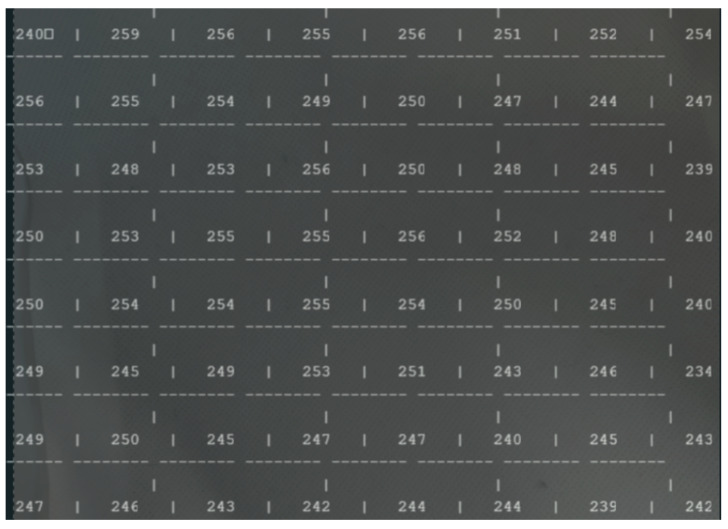
Results of the ToF sensor with empty office trash box.

**Figure 14:**
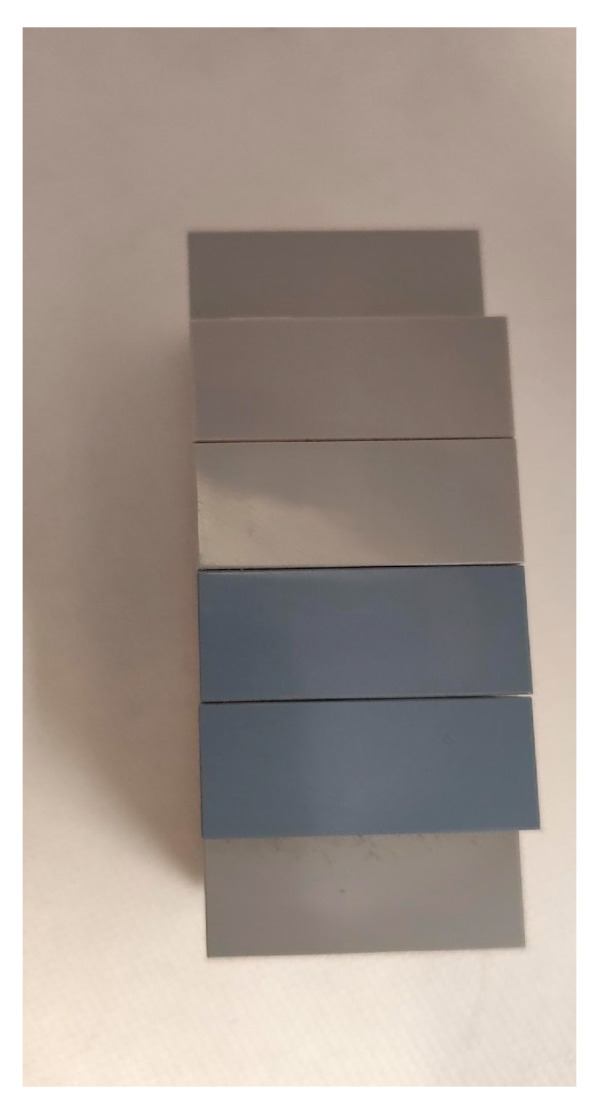
First block added inside the office trash box.

**Figure 15:**
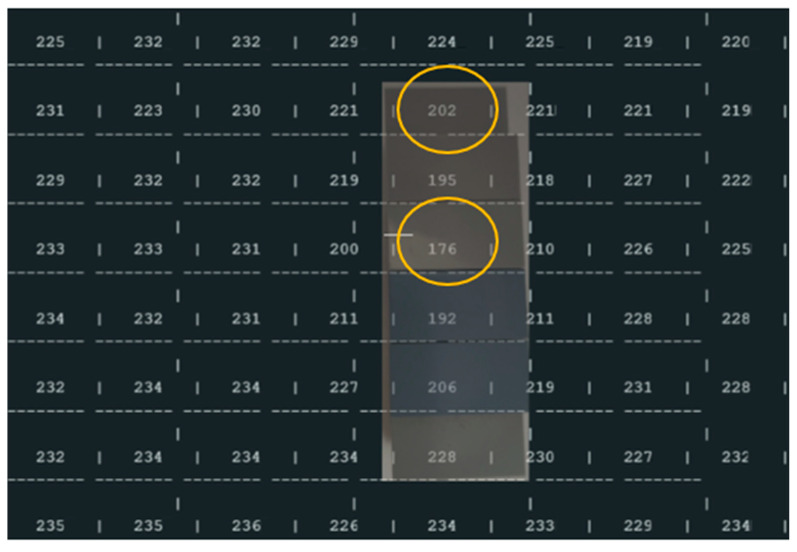
Results of the ToF sensor with the first block inside the office trash box (superposed images), yellow circle experimental values used to compare with real values.

**Figure 16:**
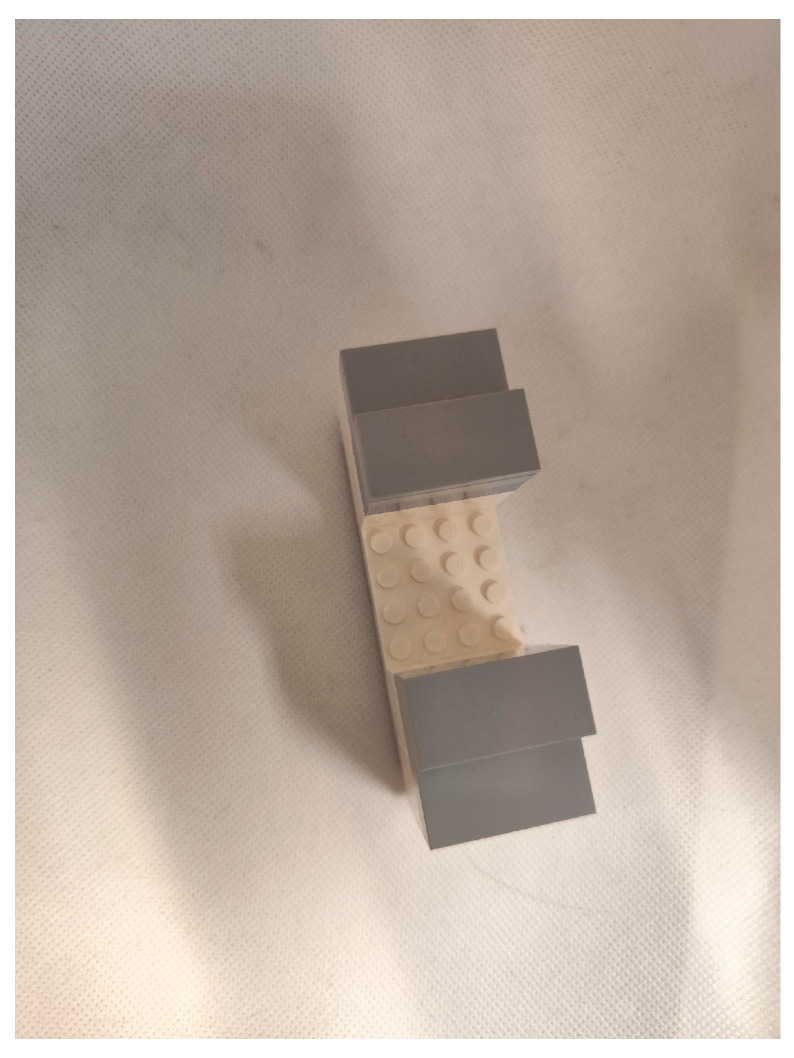
Second block (with new block dimensions) added inside the office trash box.

**Figure 17:**
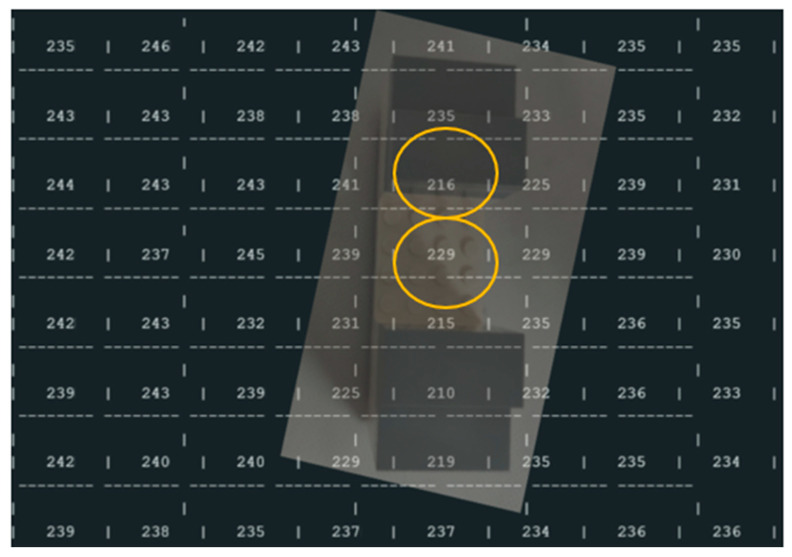
Results of the ToF sensor with the new dimensions block inside the office trash box, yellow circle experimental values used to compare with real values.

1. Measuring Height with ToF Sensor (Figure 12 and Figure 13): This experiment involves using the ToF sensor to measure the height of objects. It sets the foundation for understanding the sensors capabilities and accuracy.
2. Adding a Block (Figure 14 and Figure 15): In this scenario, a block is introduced to the setup. The goal is to observe how the sensor measurements are affected by the added structure.
3. New Block Dimensions (Figure 16 and Figure 17): This scenario introduces new block dimensions and analyzes the impact on sensor readings. It helps in refining the measurement process and understanding the sensor’s response to different object sizes.

These scenarios collectively will provide a comprehensive understanding of the ToF sensor performance in various conditions and setups.

In the next sections, we will provide the metrics of each block achieved with the help of calipers.

#### 4.1.1. Scenario: Measuring Height with the ToF Sensor

In this experiment (Figure 12), we have an empty photography block to understand the main height we will work with during this process. We then add a block to observe its effect. The study follows this structure: description, demonstration of the physical scenario, and overlay of the scenario with the values of the results.

Initial Setup:

- Empty Photography Block: The initial setup involves an empty photography block to measure the main height.
- Measurement: With the ToF sensor in the top of the office trash, the maximum height is 250 mm (25 cm, corresponding to the height of the office trash, Figure 11).

Figure 13 presents the “view” of the ToF sensor.

Analyzing the results shown in Figure 13, it is possible to observe an average of 250 mm (the ToF sensor measures in mm), that is, 25 cm from the top to the base of the office trash box, shown at the beginning (Figure 12).

#### 4.1.2. Adding a Block

- Block dimensions: A block (piece) with a height of approximately 25 mm is added (overlapped) to the base block (another piece) of 52 mm inside the office trash box (Figure 14). This block is smaller than the base block.
- Experimental results (Figure 15):

Practical Measurement (base of the block, yellow circle): 250 mm − 202 mm = 48 mm

Difference: 52 − 48 mm = 4 mm

Practical Measurement (Top of block): 250 mm − 176 mm = 74 mm

Difference: 77 (52 + 25) mm − 74 mm (real) = 3 mm

#### 4.1.3. New Block Dimensions (Two Different Blocks-Figure 16)

○ Base to Top of Object: 36 mm
○ Base to Hole: 20 mm

- Experimental results (Figure 17, yellow circle):
  ○ Base to Top of Object: 250 mm − 216 mm = 34 mm
    ▪ Difference: 36 − 34 mm = 2 mm
  ○ Base to Hole: 250 mm − 229 mm = 21 mm
    ▪ Difference: 20 − 21 mm = −1 mm

To evaluate and analyze the data collected, a table (Table 2) was created that summarizes the results. Note the following:

- Negative Measurements: Indicate that the sensor perceives the object as lower than it is.
- Positive Measurements: Indicate that the sensor perceives the object as higher than it is.
- Value 0: Does not exist in the measurements.

Reflections can sometimes cause discrepancies in measurements. However, these reflections generally do not significantly impact the overall precision of the system. Additionally, this system is not intended for use in garbage cans containing glass or metal, which helps mitigate potential issues from reflections.

### 4.2. Scenario in a Closed Box

**Figure 18:**
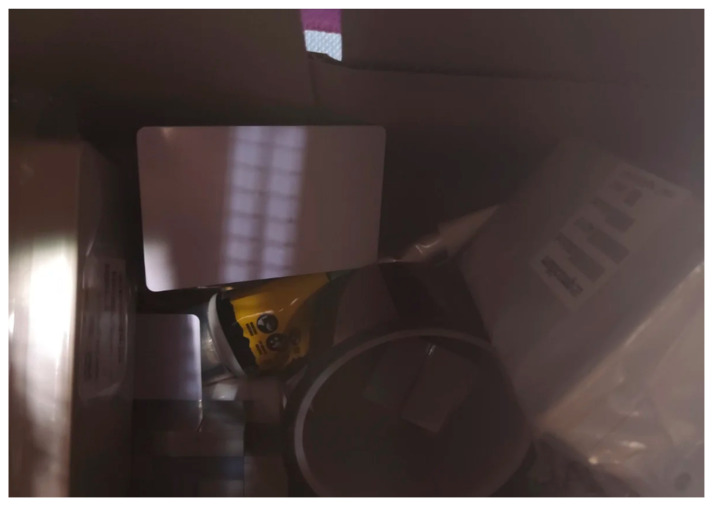
Different kinds of blocks in the inside the office trash box.

**Figure 19:**
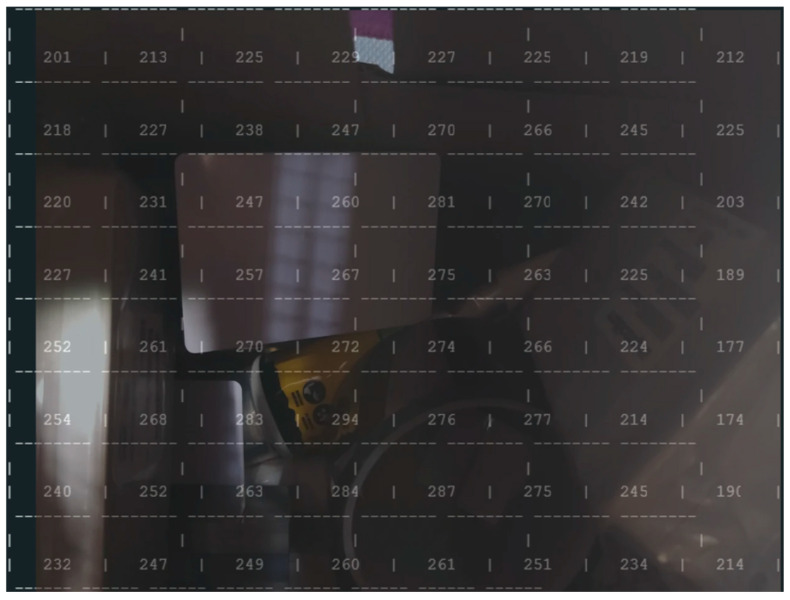
Results of different kinds of blocks in the inside of the office trash box.

In this scenario (Figure 18), multiple objects were stacked inside a closed box to simulate a confined environment, and the ToF-Node stands at a height of 25 cm. This setup allows for detecting the relative quantity of trash in a trash can, for example, testing the sensor’s ability to detect and analyze objects in a restricted space. It helps perceive inclinations and various possible reliefs through the values received, despite the irregular angles of the objects preventing exact measurements. The results are presented in Figure 19.

The maximum recorded value is 294 mm. The objects are closer to the sensor (200 to 300 mm) and closer together. The arrangement of the values suggests that the sensor is measuring an uneven surface or an object with depth variations. Some values are quite different from other experiences due to different reflections and the objects being very close together.

Following the results shown in Figure 19, we showed the ToF-Node taking measurements over periods of 30 min, 2 h, and 24 h. The results can be seen in Table 3, Table 4 and Table 5.

Table 3 displays the measurements taken by the ToF-Node during a 30-min period. Each column represents a different measurement point, and each row shows the recorded values at various intervals. The data indicate consistent readings across the measurement points, with slight variations that are within acceptable limits for real-time monitoring.

Table 4 presents the measurements taken by the ToF-Node over a 2-h period. Like Table 3, each column represents a different measurement point, and each row shows the recorded values at various intervals. The data show a high level of consistency, with minor deviations that do not significantly impact the overall accuracy of the system.

Table 5 provides the measurements taken by the ToF-Node during a 24-h period. The recorded values are shown in each column and row, indicating the system’s ability to maintain accuracy over extended periods. The data demonstrate the robustness of the ToF-Node, with consistent readings and minimal deviations.

Table 6 presents the average variance recorded for each point (row vs. column) during the 30-min, 2 h, and 24-h measurement periods.

The average variance values in Table 6 are calculated by comparing the measurements in each row and column across Table 3, Table 4 and Table 5. These values indicate how much the recorded measurements deviate from the expected values, with lower variance values representing higher precision and accuracy.

- Column 1: The variance values range from 0.0 to 0.4, indicating minimal deviation across the measurement points. This consistency demonstrates the system’s ability to maintain accurate readings over different time periods.
- Column 2: The variance values range from 0.18 to 0.42, showing slightly higher deviations compared to Column 1. However, these values are still within acceptable limits, ensuring reliable data collection.
- Column 3: The variance values range from 0.2 to 0.4, indicating consistent accuracy across the measurement points. The system effectively handles variations in object dimensions and positions.
- Column 4: The variance values range from 0.17 to 0.44, demonstrating the system’s robustness in maintaining precision over extended periods.
- Column 5: The variance values range from 0.0 to 0.38, showing minimal deviations and high accuracy in the recorded measurements.
- Column 6: The variance values range from 0.0 to 0.46, indicating slightly higher deviations but still within acceptable limits for real-time monitoring applications.
- Column 7: The variance values range from 0.0 to 0.53, showing the highest deviations among the columns. However, these values are still acceptable for ensuring reliable data collection.
- Column 8: The variance values range from 0.0 to 0.46, demonstrating consistent accuracy across the measurement points.

The average variance values in Table 6 provide a comprehensive overview of the system’s precision and accuracy. The deviations recorded are minimal, with an overall average variance of approximately 0.5%, which is within acceptable limits for real-time monitoring applications. This level of precision is crucial for ensuring reliable data collection and analysis, validating the effectiveness of the ToF-Node for real-time monitoring applications.

### 4.3. Cloud Scenario

**Figure 20:**
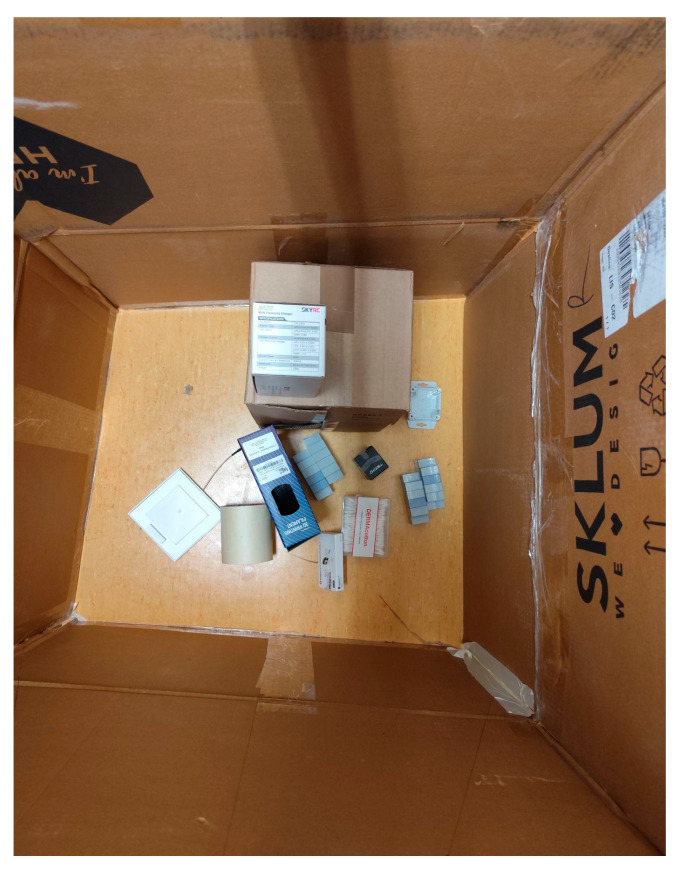
Biggest box: different kinds of blocks in the inside the office trash box.

Finally, we try another experience to send data collected to IoT cloud. In Figure 20, we present the trash inside the box with the biggest box. The ToF-Node stands at a height of 110 cm.

**Figure 21:**
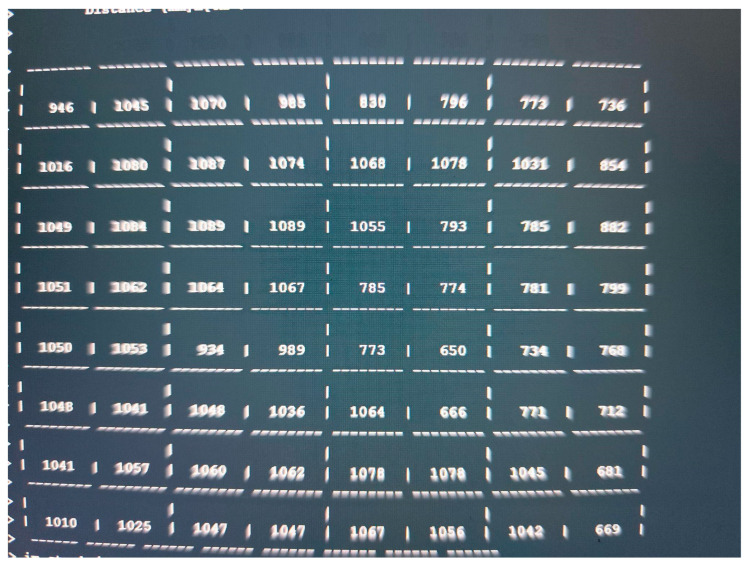
Results: biggest box: different kinds of blocks in the inside of the office trash box.

The results are presented in Figure 21.

Smaller values (e.g., 650, 666, 681 mm) indicate the presence of a closer object in the area. The larger values (e.g., 1089, 1078, 1068 mm) represent more distant areas or flat surfaces. The arrangement of the values suggests that the sensor is measuring an uneven surface or an object with depth variations. The maximum values recorded in the 8 × 8 matrix were on the order of 1089 mm (approximately 1.09 m). There was significant variation between different areas of the matrix. Looking at Figure 20, in the top left-hand corner you can see a value of 946, and in the opposite corner, you can see a value of 736. However, if we analyze Figure 19, there are no objects in these two corners. These results are due to the reflection on the walls of the office trash box, because the box used is small. This leads us to think that in future experiments, we should have a larger office trash box, but in a real situation, this aspect would not occur, and if it did, we should proceed to configure the range of the sensor, as well as its position on the top of the trash container.

**Figure 22:**
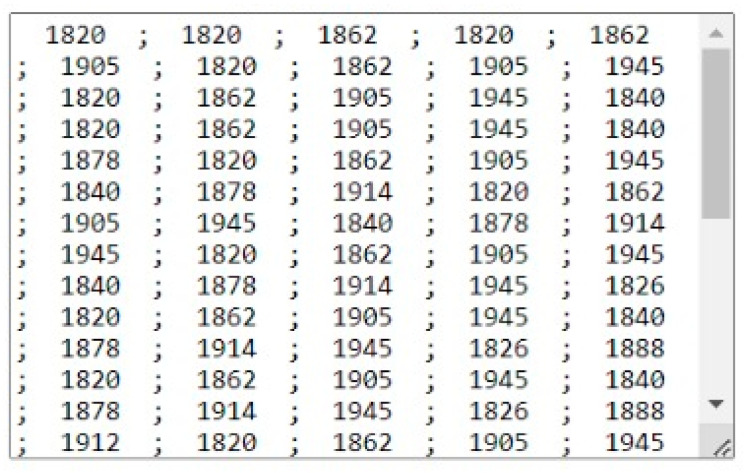
Data formats send to the IoT gateway cloud.

In Figure 22, we present the data sent to the IoT gateway (RP3)

**Figure 23:**
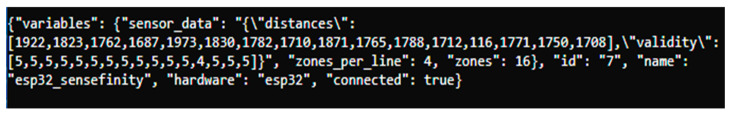
Data formats are present in the IoT cloud in a text format.

The API of the Sensefinity IoT cloud lets us access an IP address, and, through the HTTP service using port 80, we can access the values that we wish using a User Interface (UI). We also added the reliability of the values on a scale from 0 to 5; this allows us to know if it is a good idea to use that value, or if that value is reliable for measurements or decisions to be made according to those same values. For instance, if the value has a reliability of “5”, then it means that it is a strong value, a strong signal that we can count on, while if it goes down to a 2, it means that the value might be inconsistent and that we should not really trust it. Once we introduce the IP address followed by the gate, (e.g.,: xxx.xxx.x.xxx:yyyy) we get what the API is sending or representing; Figure 23.

**Figure 24:**
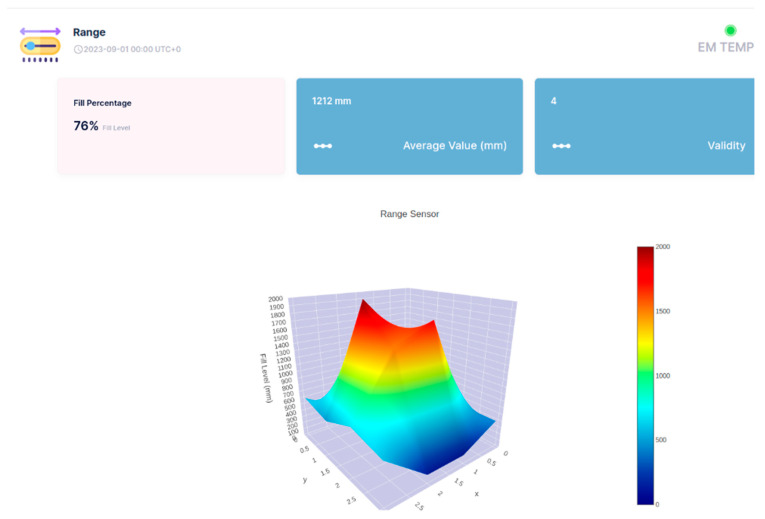
3D representation through Sensefinity cloud—view 1.

**Figure 25:**
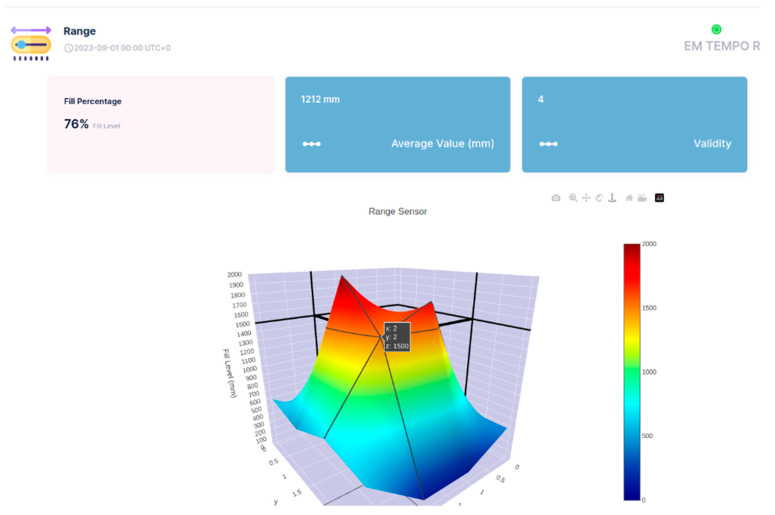
3D representation through Sensefinity cloud—view 2.

**Figure 26:**
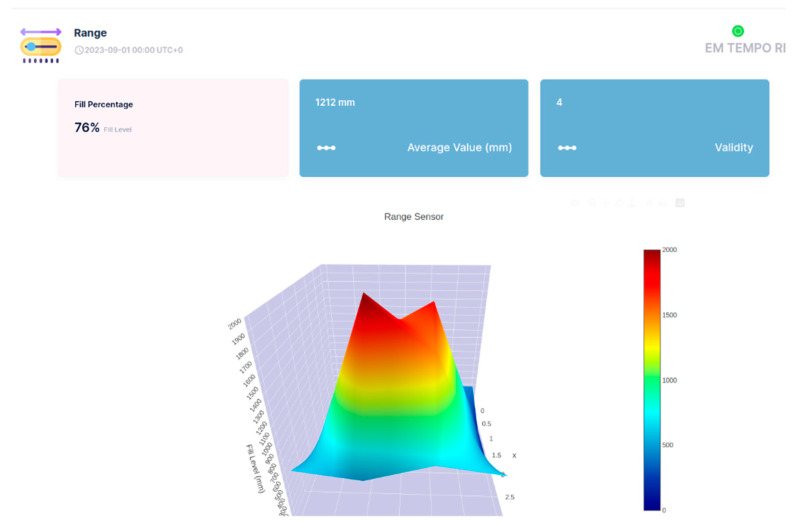
3D representation through Sensefinity cloud—view 3.

**Figure 27:**
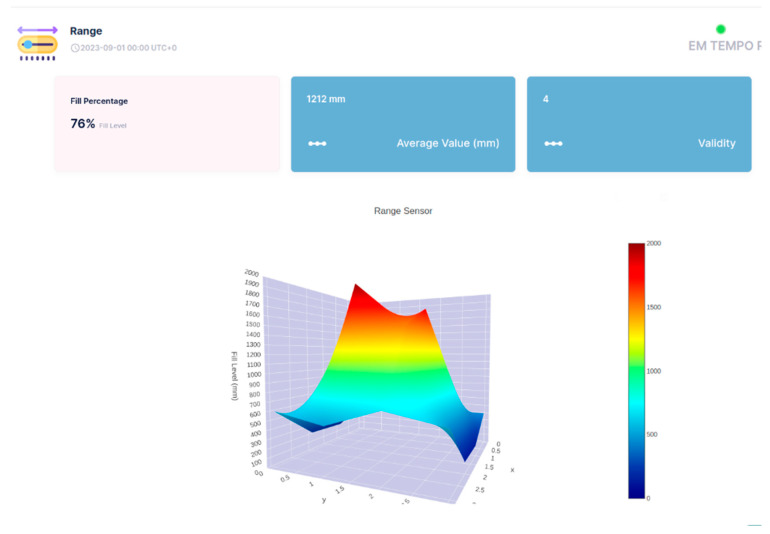
3D representation through Sensefinity cloud—view 4.

So, this is all about a potential conception and adoption of API, which we hope to use for a 3D visualization of the objects inside the container, using the cloud processing aptitude of Sensefinity to know where and how high they are positioned (see Figure 24, Figure 25, Figure 26 and Figure 27). For example, with this API, we can export the data via Wi-Fi and get the 3D rendering of the values in the cloud storage, allowing us to monitor the space.

In Figure 24, Figure 25, Figure 26 and Figure 27, the interface belongs to Sensefinity and displays data related to the volumetry of a tank or container. Interface elements:

- Fill (%)—Indicates an occupancy level of 76%, referring to the fraction of the container’s total volume.
- Average Value (mm)—The average of the measurements taken by the sensor is 1212 mm.
- Validity—An indicator of data quality, in this case represented by the value 4.
- 3D Graph—Volumetric representation of sensor data, showing a colored surface with a depth scale. The coloring varies from blue (low level) to red (high level), making it possible to visualize irregularities on the surface of the measured material.
- Real-time update—The interface displays the information with continuous updates to allow dynamic monitoring of the values.

For example, in Figure 25, highlighted in the 3D Graph, the volumetric graph now displays a specific point with the coordinates: X: 2; Y: 2; Z: 1500 mm.

This represents a point in three-dimensional space where the sensor has measured a depth of 1500 mm.

Improved Visualization: The inclusion of black lines in the graph improves the segmentation and interpretation of the depth data.

Rest of Interface: The values displayed remain the same: 76% full, 1212 mm average value, and Validity of 4.

This type of detailed visualization helps to identify patterns, variations in the volumetry of the measured material, and IoT applications that require volumetric monitoring, such as tank, silo, or waste container management, enabling logistics optimization and decision-making based on real-time data.

### 4.4. Energy Consumption

The VL53L8CX ToF sensor and ESP32-S3 microcontroller communicate in different power consumption modes to maximize battery life. The VL53L8CX requires 10 mA in active mode and only 55 µA in low-power mode. The ESP32-S3 consumes 45 mA when not using Wi-Fi and 120 mA while transmitting data over Wi-Fi, and can achieve deep sleep with a current draw of just 100 µA.

Data transmission is split into two stages: 30 ms for communication from the ESP32-S3 to the gateway and 200 ms for the gateway to the cloud. One data acquisition cycle lasts 15 ms per frame, with each frame consisting of depth data measurements from 64 points to either rebuild the depth image or analyze the environment. A Joulescope was used to measure consumption accurately.

Every 3600 s, measuring and sending data consumes 130 mA for 45 ms (15 ms measurement + 30 ms Wi-Fi transmission), resulting in an energy expenditure of 5.85 mAh. During the remaining 3599.955 s, the system is in low-power mode, consuming 0.558 mAh, given by the combined consumption of the ESP32-S3 and ToF sensor at 155 µA.

Repeating this measuring and sending cycle every 3600 s results in 24 cycles per day, leading to a total daily consumption of approximately 153.8 mAh. Considering a 3.6 V, 3350 mAh Li-Ion 18650 battery (LG INR18650 model), the system’s estimated autonomy is around 22 days.

Compared to other technologies, LiDAR or RFID, in terms of consumption, LiDAR technology, in general terms, analyzed manufacturers’ datasheets, such as [21], LIDAR-Lite v3 105 mA idle and 130 mA continuous. RDIF technology, in this case the reader with the highest current consumption and according to manufacturer datasheets, such as [22], has consumption as follows: UHF RFID Readers: 1 W to 20 W (200 mA to 4 A at 5 V); passive UHF RFID Tags: ~1 µW to 10 µW (negligible current until activated); active UHF RFID Tags: 10 mW to 100 mW (10 µA to 100 mA). Therefore, according to the experience shown in this manuscript, the use of the ToF sensor solution is clearly viable in terms of consumption.

### 4.5. Research Contributions and Limitations

Our research contributes to the field in several significant ways, namely:

Precision and Accuracy: We conducted detailed experiments to evaluate the precision and accuracy of our system. Table 3, Table 4 and Table 5 summarize the results of the ToF-Node measurements taken over periods of 30 min, 2 h, and 24 h. Table 6 shows the average variance recorded for each point, indicating a deviation of approximately 0.5%. This level of detailed analysis and the specific focus on ToF sensors for real-time monitoring is unique to our work.

Three-Dimensional Visualization: The three-dimensional visualization of volume and level on the cloud IoT dashboard facilitates the incorporation of additional ToF sensors, reducing the importance of sensor placement within waste containers. This advancement ensures precise and consistent data for essential decision-making.

Future Potential with Blockchain: There is potential for data storage on a blockchain platform, enabling the monitoring of waste collection and delivery routes. This capability supports cloud-based tracking of waste collection vehicles, achievable through the integration of a geolocator and communication with the cloud.

Cost-Effectiveness and Scalability: Our system is cost-effective, utilizing affordable components like the ToF sensor and ESP32-S3, making it accessible for various applications without significant financial investment. The total cost would be EUR 22. The scalability of our system, allowing for the easy addition of more sensors or devices, is also a key advantage.

Real-Time Monitoring and Reliability: The integration of IoT technologies allows for real-time monitoring of waste bins, enabling proactive scheduling of collection visits and preventing overflow. The reliability scale implemented in the API helps in assessing the trustworthiness of the data, ensuring that only accurate and consistent values are used for critical decisions. These aspects enhance the efficiency of waste collection services.

While the proposed system offers significant advancements in real-time monitoring of garbage levels using volumetric ToF sensors, there are certain limitations that need to be acknowledged:

1. Environmental Factors: The accuracy of ToF sensors can be affected by environmental conditions such as extreme temperatures, humidity, and lighting variations. These factors may introduce noise and impact the precision of measurements.
2. Sensor Placement: Optimal placement of sensors within waste containers is still necessary to ensure accurate reading. Incorrect placement may lead to inconsistent data.
3. Data Transmission: The reliance on Wi-Fi networks for data transmission may pose challenges in areas with poor connectivity, affecting the real-time monitoring capabilities of the system.
4. Real-World Testing: The system has been primarily tested in laboratory conditions. Real-world testing in diverse environments, such as public residential areas, is necessary to validate its effectiveness and reliability.

By acknowledging these limitations, we aim to provide a balanced view of the proposed approach and highlight areas for future improvement and research.

## 5. Conclusions

This study presents an integrated approach resulting in a prototype that offers a reliable and affordable solution for real-time monitoring of garbage levels using volumetric ToF sensors. Initially, the system was designed to connect to a Wi-Fi network, with the Raspberry Pi functioning as a gateway with cloud access and the ESP32-S3 gathering local data. The data were transmitted securely to the cloud using the HTTP protocol, enabling efficient communication between the local device and the remote cloud infrastructure.

The findings are quite promising, as laboratory tests demonstrated a higher level of precision than would be necessary in real-world conditions. This suggests that the system is robust and effective even under less-controlled environments. The decision to incorporate the ToF-Node has proven advantageous, enhancing usability and confirming its low power consumption, which underscores its portability.

The three-dimensional visualization of volume and level on the cloud IoT dashboard has demonstrated significant advantages, facilitating the prospective incorporation of additional ToF sensors. This advancement will reduce the importance of sensor placement within waste containers. Additionally, the reliability scale integrated into the API aids in evaluating the credibility of the data, ensuring that only precise and consistent information is utilized for essential decision-making. In the future, there is potential for data storage on a blockchain, such as an elastic blockchain platform, which would enable the monitoring of waste collection and delivery routes. This capability would support cloud-based tracking of the permanent location of waste collection vehicles, achievable through the straightforward integration of a geolocator and communication with the cloud.

The system proposed also has several other advantages. The use of a RESTful API and HTTP protocol ensures compatibility with various cloud platforms and services, providing flexibility in data handling and processing. The low power consumption of the ToF-Node highlights its complete portability, making it suitable for various environments and applications. Additionally, the small size of the ToF-Node (40 × 18 × 4 mm PCB) and its enclosure (65 × 40 × 30 mm) make it easy to deploy in various urban environments.

The ToF sensor’s high precision revealed itself to be essential for accurately measuring and detecting variations in object dimensions and positions. This ensures reliable data collection and analysis, which is fundamental for real-time monitoring and decision-making processes. In this experiment, the objects modeled a trash bin, demonstrating the sensor’s ability to detect the fill level, identify irregularities, and monitor the overall status of the bin. This capability enhances the system’s effectiveness in various environments, making it suitable for applications such as waste management, inventory control, and environmental monitoring.

Overall, this solution provides a comprehensive, reliable, and scalable approach to monitoring and data transmission, making it suitable for a wide range of applications that can be applied, particularly in the context of smart cities. The successful integration of ToF sensors in a connected device ecosystem, along with the effective use of cloud services, demonstrates its potential to enhance real-time monitoring and decision-making processes. Additionally, the system can be integrated into a multi-parameter monitoring setup, where not only fill levels but also humidity, temperature, and harmful gases can be monitored. This would be the object of analysis in future work. Conversely, it will be important to consider the management of various ToF-Nodes at the gateway level in the future, as well as conducting tests outside of laboratory settings, specifically in waste containers utilized in public residential areas.

## Figures and Tables

**Table 1:** Summary of the relevant research discussed.

Article	Technologies Used	Focus	Sensor Used
[12]	Only simulation	IoT-based platforms addressing urban waste collection optimization	Ultrasonic and gas
[13]	LoRaWAN, ZigBee, Wi-Fi, GSM, and Cloud	Examines the cyber and physical threats affecting SWMSs	Ultrasonic, temperature, and gas
[14]	Robotic arm and Computational vision	Influence pertaining to robotics on intelligent urban waste management with a focus on automation in collection, sorting, recycling, and disposal	Ultrasonic
[16]	LiDAR,	Experimental tests within indoor	LiDAR
This work	Wi-Fi, and IoT Cloud	Real-time monitoring system for urban garbage levels	ToF

**Table 2:** Summary of the experiments.

Scene	Values Measured by Caliper (mm)	Practical Values (mm)	Difference (mm)
Adding a Block (Section 4.1.2, base of the block)	52	48	4
Adding a Block (Section 4.1.2, Top of block)	77	74	3
New Block Dimensions (Section 4.1.3, Top of object)	36	34	2
New Block Dimensions (Section 4.1.3, Base to hole)	20	21	−1

**Table 3:** ToF-Node measuring for 30 min.

# Line	Column 1	Column 2	Column 3	Column 4	Column 5	Column 6	Column 7	Column 8
1	201.0	213.0	225.0	227.0	225.0	219.0	212.0	218.0
2	227.0	238.0	247.0	270.0	266.0	245.0	225.0	220.0
3	231.0	247.0	260.0	281.0	270.0	242.0	203.0	227.0
4	241.0	257.0	267.0	275.0	264.0	225.0	189.0	252.0
5	261.0	270.0	272.0	274.0	266.0	224.0	177.0	254.0
6	268.0	283.0	294.0	276.0	277.0	214.0	174.0	240.0
7	252.0	263.0	284.0	287.0	275.0	245.0	190.0	249.0
8	232.0	247.0	249.0	260.0	261.0	251.0	234.0	238.0

**Table 4:** ToF-Node measuring for 2 h.

# Line	Column 1	Column 2	Column 3	Column 4	Column 5	Column 6	Column 7	Column 8
1	201.0	214.0	225.0	228.0	225.0	220.0	212.0	217.0
2	227.0	237.0	247.0	269.0	266.0	246.0	226.0	220.0
3	230.0	247.0	259.0	282.0	271.0	241.0	202.0	227.0
4	240.0	257.0	267.0	274.0	263.0	224.0	188.0	252.0
5	260.0	270.0	273.0	274.0	267.0	224.0	177.0	255.0
6	268.0	284.0	295.0	275.0	278.0	214.0	174.0	240.0
7	253.0	262.0	285.0	286.0	274.0	246.0	190.0	250.0
8	232.0	248.0	248.0	261.0	261.0	250.0	235.0	237.0

**Table 5:** ToF-Node measuring for 24 h.

# Line	Column 1	Column 2	Column 3	Column 4	Column 5	Column 6	Column 7	Column 8
1	202.0	214.0	224.0	229.0	226.0	221.0	212.0	218.0
2	226.0	236.0	246.0	270.0	266.0	247.0	227.0	220.0
3	230.0	246.0	258.0	282.0	270.0	240.0	201.0	226.0
4	240.0	257.0	266.0	274.0	263.0	223.0	189.0	253.0
5	259.0	269.0	272.0	275.0	266.0	224.0	177.0	254.0
6	268.0	284.0	294.0	274.0	278.0	214.0	175.0	239.0
7	254.0	262.0	284.0	286.0	275.0	245.0	190.0	249.0
8	232.0	248.0	247.0	261.0	261.0	250.0	234.0	236.0

**Table 6:** Average variance (each line vs. column): 30 min, 2 h, and 24 h.

# Line	Column 1	Column 2	Column 3	Column 4	Column 5	Column 6	Column 7	Column 8
1	0.25	0.23	0.22	0.44	0.22	0.46	0.0	0.46
2	0.22	0.42	0.2	0.37	0.0	0.41	0.44	0.0
3	0.22	0.2	0.39	0.18	0.37	0.41	0.49	0.22
4	0.21	0.0	0.19	0.18	0.19	0.45	0.53	0.2
5	0.38	0.19	0.37	0.18	0.38	0.0	0.0	0.39
6	0.0	0.18	0.34	0.36	0.18	0.0	0.29	0.21
7	0.4	0.19	0.35	0.17	0.36	0.41	0.0	0.4
8	0.0	0.2	0.4	0.19	0.0	0.2	0.43	0.42

## Data Availability

Data is contained within the article.

## References

[1] United Nations (2023). Sustainable Development Goals (SDGs). https://sdgs.un.org/goals.

[2] European Commission (2015). Circular Economy Package. https://ec.europa.eu/environment/circular-economy/.

[3] European Commission (2018). Strategy for Plastics in the Circular Economy. https://ec.europa.eu/environment/waste/plastic_waste.htm.

[4] European Commission (2020). Strategy for the Circular Economy. https://environment.ec.europa.eu/strategy/circular-economy-action-plan_en.

[5] Wang T.-L., Ao L., Zheng J., Sun Z.-B. (2023). Reconstructing Depth Images for Time-of-Flight Cameras Based on Second-Order Correlation Functions. Photonics.

[6] Qian X., Jiang W., Elsharabasy A., Deen M.J. (2023). Modeling for Single-Photon Avalanche Diodes: State-of-the-Art and Research Challenges. Sensors.

[7] He Y., Liang B., Zou Y., He J., Yang J. (2017). Depth Errors Analysis and Correction for Time-of-Flight (ToF) Cameras. Sensors.

[8] Luan X. (2001). Experimental Investigation of Photonic Mixer Device and Development of TOF 3D Ranging Systems Based on PMD Technology. Ph.D. Thesis.

[9] Li L. Time-of-Flight Camera–An Introduction. https://www.ti.com/lit/wp/sloa190b/sloa190b.pdf.

[10] Streeter L. (2018). Time-of-Flight Range Image Measurement in the Presence of Transverse Motion Using the Kalman Filter. IEEE Trans. Instrum. Meas..

[11] Hansard M., Lee S., Choi O., Horaud R. (2012). Time of Flight Cameras: Principles, Methods, and Applications.

[12] Khan S., Ali B., Alharbi A.A.K., Alotaibi S., Alkhathami M. (2024). Efficient IoT-Assisted Waste Collection for Urban Smart Cities. Sensors.

[13] Brighente A., Conti M., Di Renzone G., Peruzzi G., Pozzebon A. (2024). Security and Privacy of Smart Waste Management Systems: A Cyber–Physical System Perspective. IEEE Internet Things J..

[14] Sutikno, Fathurrahman H.I.K., Wahono T., Handayani L. (2024). Robotics Technologies in Urban Smart Waste Management. High-Tech. Innov. Ser..

[15] Zoumpoulis P., Konstantinidis F.K., Tsimiklis G., Amditis A. (2024). Smart Bins for Enhanced Resource Recovery and Sustainable Urban Waste Practices in Smart Cities: A Systematic Literature Review. Cities.

[16] Zhang Y., Chen J., Wang Q. (2022). Time-of-Flight Sensors vs. LiDAR: A Comparative Study for Indoor Mapping Applications. Sensors.

[17] STMicroelectronics VL53L8CX. https://www.st.com/en/imaging-and-photonics-solutions/vl53l8cx.html.

[18] Espressif Systems ESP32-S3. https://www.espressif.com/en/products/socs/esp32-s3.

[19] Raspberry Pi Raspberry Pi 3–Model B. https://www.raspberrypi.com/products/raspberry-pi-3-model-b/.

[20] Sensefinity https://www.sensefinity.com/.

[21] LiDAR. https://www.arizontw.com/proimages/product/Reader_AL-510_datasheet_.pdf.

[22] RFID. https://static.garmin.com/pumac/LIDAR_Lite_v3_Operation_Manual_and_Technical_Specifications.pdf.

